# Role of high-mobility group box-1 in myocardial ischemia/reperfusion injury and the effect of ethyl pyruvate

**DOI:** 10.3892/etm.2015.2290

**Published:** 2015-02-13

**Authors:** YUNLING LIN, LIANGLONG CHEN, WEIWEI LI, JUN FANG

**Affiliations:** Department of Cardiology, Fujian Medical University Union Hospital, Fuzhou, Fujian 350000, P.R. China

**Keywords:** high-mobility group box-1, ethyl pyruvate, ischemia/reperfusion injury, inflammation

## Abstract

High-mobility group box-1 (HMGB1) acts as a proinflammatory cytokine that triggers and amplifies the inflammation cascade following ischemia/reperfusion (I/R). Ethyl pyruvate (EP) has been reported to inhibit HMGB1 release in several I/R models. This study was designed to investigate the potential role of HMGB1 in a rat myocardial I/R model and to determine the effect of EP. Male Sprague Dawley rats were subjected to 30 min myocardial ischemia and 48 h reperfusion. In protocol 1, the rats were assigned to one of four groups (n=16 per group): Phosphate-buffered saline (PBS) or recombinant human HMGB1 (rhHMGB1) at three different doses (1, 10 or 100 μg/kg). In protocol 2, the rats were also assigned to one of four groups (n=16 per group): Sham, control, EP and EP + rhHMGB1. EP (40 mg/kg) or rhHMGB1 (100 μg/kg) was injected intravenously prior to reperfusion. Hemodynamic measurements were performed, and myocardial infarct size (IS) was calculated. Western blotting was conducted to evaluate HMGB1, tumor necrosis factor-α (TNF-α) and interleukin-6 (IL-6) expression levels. In the protocol 1 rats, the IS was markedly increased in the rhHMGB1 (100 μg/kg) group compared with that in the PBS group, and this increase was accompanied by elevated levels of TNF-α and IL-6. In the protocol 2 rats, I/R resulted a 4.8-fold increase in HMGB1 expression with an increased IS and impaired cardiac function compared with the sham group. EP significantly inhibited the elevated HMGB1 level, suppressed the activated TNF-α and IL-6 and reduced cardiac dysfunction. This cardioprotection was abolished by rhHMGB1. In conclusion, accumulation of HMGB1 is deleterious to the heart following myocardial I/R. EP can exert a strong protective effect against myocardial I/R injury, and these benefits are associated with a reduction in HMGB1.

## Introduction

For patients with acute myocardial infarction, early reperfusion of the ischemic myocardium, either through thrombolytic therapy or primary percutaneous coronary intervention, is the most effective treatment to limit the infarct size (IS) and improve the clinical outcome; however, the process of restoring blood flow to the ischemic myocardium has the potential to induce additional lethal injuries and reduce myocardial salvage. Inflammatory cells and cytokines play an important role in the pathogenesis of myocardial reperfusion injury, not only by triggering deleterious responses but also by amplifying ongoing responses to build a cascade of injury (1). Numerous experimental studies have demonstrated the efficacy of anti-inflammation therapies against myocardial ischemia/reperfusion (I/R); however, the translation of these beneficial effects into the clinical setting has been disappointing (1,2). The efficacy that has been shown for most anti-inflammatory agents in animal models has been difficult to confirm in clinical trials. The trigger and the mechanism of inflammation propagation warrant further studies.

It has previously been shown that high-mobility group box-1 (HMGB1) is a potent innate ‘danger signal’. HMGB1 has been demonstrated to initiate and amplify the inflammatory response following I/R injury (3,4,5). Recent studies have shown that HMGB1, acting via the receptor for advanced glycation end products (RAGE) or members of the Toll-like family of receptors (TLRs), is an early mediator of organ damage in myocardial I/R injury (4,5). Blockage of HMGB1 with HMGB1 box A, a specific antagonist of whole HMGB1 on the RAGE receptor, or anti-HMGB1 monoclonal antibody has been proved to exert benefit against I/R injury (6,7); however, these antagonists are expensive and their source is limited. Ethyl pyruvate (EP), the first described pharmacological inhibitor of HMGB1 secretion (8), has been revealed to protect various organs against I/R injury *in vivo*, including the kidney and liver (9,10). The aims of the present study were to investigate the role of HMGB1 in a myocardial I/R model, to explore the association between HMGB1 and the inflammatory mediators and to determine the benefit of EP.

## Materials and methods

### Animal care

Male Sprague Dawley rats (220–250 g) were purchased from the National Rodent Laboratory Animal Resources, Shanghai Branch (Shanghai, China). The animals were housed under standard conditions, maintained under a diurnal 12-h light/dark cycle and fed standard rat food and water *ad libitum*. The present study was performed in accordance with the guidelines of the National Institutes of Health (NIH) Guide for the Care and Use of Laboratory Animals (NIH publication no. 85–23, revised 1996) and was approved by the Animal Care and Use Committee of Fujian Medical University (Fuzhou, China).

### In vivo myocardial I/R model

The rats were anesthetized with pentobarbital sodium (50 mg/kg, intraperitoneal). Additional doses were administered as required to maintain anesthesia. The rats were intubated and ventilated with 100% oxygen. The chest was opened via a left thoracotomy through the fourth or fifth intercostal space and the hearts were exposed. An 8-0 silk ligature was placed under the left coronary artery (LCA) and tied using a shoestring knot. Myocardial ischemia was confirmed by the presence of discoloration of the ischemic area, left ventricular (LV) dyskinesia and ST-segment elevation on an electrocardiogram. Following occlusion for 30 min, reperfusion was initiated by releasing the knot. Reperfusion was confirmed by the return of color to the ischemic area. The loosened suture was left in place and then retied for the purpose of evaluating the ischemic area. The chest wall was closed, the animal extubated and body temperature maintained by use of a 37°C warm plate. In the sham-operated animals, the same procedure was performed with the exception of the coronary artery ligation. The rats were sacrificed and the hearts were harvested 48 h after reperfusion.

### Treatment preparation

EP (Sigma-Aldrich, St. Louis, MO, USA) was dissolved in a Ringer’s lactate solution (RLS; TianRui Pharmaceutical Group Co., Ltd., Rui’an, China) containing sodium (130 mmol/l), potassium (4 mmol/l), calcium (2.7 mmol/l) and chloride (139 mmol/l) ions (pH 7.0). EP was administered intravenously and the injection volume was 0.5 ml per dose. Recombinant human HMGB1 (rhHMGB1; BD Biosciences, Franklin Lakes, NJ, USA) was diluted with phosphate-buffered saline (PBS) with a total volume of 0.5 ml.

### Protocol assignment

The rats were divided into two groups, protocol 1 and protocol 2. The aim of protocol 1 was to determine the effect of HMGB1 on myocardial reperfusion injury *in vivo*. The rats in the protocol 1 group were further assigned to one of four groups (n=16 per group): rhHMGB1 (three doses) or PBS. After 30 min myocardial ischemia, rhHMGB1 (1, 10 or 100 μg/kg) was injected intravenously 1 min prior to reperfusion; 0.5 ml PBS was used as a vehicle in the vehicle group.

The aim of protocol 2 was to determine the effect of EP on cardiac function. The rats assigned to protocol 2 were further subdivided into four groups (n=16 per group): i) Sham (surgical procedures were performed but ischemia was not applied); ii) Control (0.5 ml RLS and 0.5 ml PBS were coadministered intravenously 2 min prior to reperfusion); iii) EP (40 mg/kg EP was administered intravenously 2 min prior to reperfusion); iv) EP + rhHMGB1 (40 mg/kg EP and 100 μg/kg rhHMGB1 were coadministered intravenously 2 min prior to reperfusion).

### Determination of LV area at risk (AAR) and IS

At the end of the 48-h reperfusion period, the LCA was religated at the original site, and 1 ml 2% Evans blue dye (Sigma-Aldrich) was injected via the external jugular vein. The heart was quickly excised and the atria, right ventricle and fatty tissues were removed. The left ventricle was sliced transversely into 4–5 slices (~2-mm). The sections were then incubated in PBS containing 2% triphenyltetrazolium chloride (Sigma-Aldrich) at 37°C for 20 min. The AAR was shown by an absence of staining with Evans blue. The AAR was separated from the nonischemic myocardium and incubated in a 37°C 1% solution of buffered (pH 7.4) triphenyltetrazolium chloride for 15 min. The AAR was then stored in vials of 10% formaldehyde overnight, and the infarcted myocardium was dissected from the AAR. The IS, AAR and LV weight were determined gravimetrically. The AAR was expressed as a percentage of the LV weight (AAR/LV weight), and the IS was expressed as a percentage of the AAR (IS/AAR) (11).

### LV function measurements. In vivo

cardiac hemodynamic function was measured with an LMS-2B dual-trace physiological recorder (Chengdu Instrument Factory, Chengdu, China) 48 h after reperfusion. The rats were anesthetized with 2% isoflurane; a micromanometer was inserted into the right carotid. Heart rate and the peak LV systolic pressure (LVSP) and LV end-diastolic pressure were measured using this catheter advanced into the LV cavity; the maximal slopes of systolic pressure increase (+dp/dt max) and diastolic pressure decrease (−dp/dt max) were analyzed.

### Western blot analysis

The total protein was extracted from the AAR of the heart. Nuclear and cytoplasmic proteins were isolated using a Nuclear Extraction kit from Active Motif, Inc. (Carlsbad, CA, USA) according to the manufacturer’s instructions. Extracts were frozen in liquid nitrogen and stored at −80°C for subsequent western blot analyses. The protein concentration was quantified using a protein assay kit from Bio Rad Laboratories (Hercules, CA, USA). Cytoplasmic or nuclear extracts (50 μg) were mixed with 2X sodium dodecyl sulfate (SDS) sample buffer and separated via 15% [for HMGB1, tumor necrosis factor-α (TNF-α) and interleukin-6 (IL-6)] SDS-polyacrylamide gel electrophoresis. The separated proteins were transferred onto Hybond™ enhanced chemiluminescence membranes (GE Healthcare Life Sciences, Pittsburgh, PA, USA). The membranes were then incubated at room temperature for 1 h with 5% skimmed milk in Tris-buffered saline containing 0.1% Tween 20 (TBST) in order to block nonspecific reactions. Following blocking, the membranes were incubated overnight with primary antibody. The primary antibodies used were as follows: Rabbit polyclonal anti-HMGB1 (1:1,000 dilution; #ab191583; Abcam, Cambridge, MA, USA), mouse monoclonal anti-TNF-α (1:1,000 dilution; #ab1793; Abcam) and mouse monoclonal anti-IL-6 (1:2,000 dilution; #MAB406; R&D Systems, Inc., Minneapolis, MN, USA). Subsequent to washing with TBST, the membrane was incubated for 1 h in 5% skimmed milk and secondary antibody (goat anti-rabbit or goat anti-mouse; 1:2,500; Abcam) conjugated to horseradish peroxidase. The immunoreactions were visualized using enhanced chemiluminescence according to the manufacturer’s instructions (Merck Millipore, Darmstadt, Germany). The protein signals were quantified using scanning densitometry, and the results from each experimental group were expressed as a relative integrated intensity compared with glyceraldehyde 3-phosphate dehydrogenase.

### Statistical analysis

Data are presented as the mean ± standard error. IS and the cardiac function parameters were analyzed using the Student’s t-test or one-way analysis of variance followed by a Least Significant Difference test. P<0.05 was considered to indicate a statistically significant difference. All statistical analyses were performed using the SPSS statistical software version 13.0 (SPSS, Inc., Chicago, IL, USA).

## Results

### Mortality and exclusion of animals

Of the 128 rats used in the study, 13 died during the surgical procedures due to bradycardia or ventricular tachycardia and three were excluded as a result of severe hypotension. Complete data sets were obtained from the remaining 112 rats.

### AAR and IS

**Figure 1:**
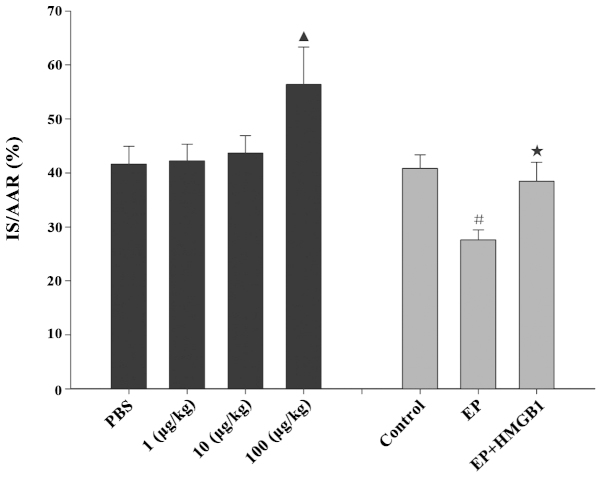
IS in rat hearts subjected to 30 min occlusion and 48 h reperfusion. PBS represents the PBS treatment group, while 1, 10 and 100 μg/kg represent the escalating concentrations of rhHMGB1 administered in the groups. The EP group was administered EP at 40 mg/kg, while the EP + HMGB1 group was administered EP at 40 mg/kg and rhHMGB1 at 100 μg/kg. Data are presented as the mean ± standard error; n=6–8 rats in all groups. Data for the sham group are not presented as the group did not undergo ischemia. ^▲^P>0.05 vs. the PBS group; ^#^P<0.05 vs. the control group; ^*^P<0.05 vs. the EP group. IS/AAR, ratio of infarct size to area at risk; PBS, phosphate-buffered saline; EP, ethyl pyruvate; rhHMGB1, recombinant human high-mobility group box-1.

The AAR/LV weight was comparable among the groups. In protocol 1, no significant difference in IS was detected among the PBS, 1 and 10 μg/kg rhHMGB1 groups (IS/AAR 41.6±3.3, 42.3±3.1 and 43.7±3.2% respectively; P>0.05, Fig. 1). Compared with the PBS group, administration of rhHMGB1 at a dose of 100 μg/kg resulted in a significant increase in IS/AAR (56.4±6.9% vs. PBS group, P<0.05, Fig. 1). In protocol 2, the IS was 40.8±2.5% of the AAR in the control group after 30 min ischemia followed by 48 h reperfusion. EP induced a significant reduction in the IS/AAR to 27.7±1.8% (P<0.05, Fig. 1); however, the addition of rhHMGB1 blocked the IS-limiting effects of EP (38.5±3.5% vs. EP group, P<0.05).

### LV function parameters

In protocol 1, no significant difference was found in the LVSP, +dp/dt max or -dp/dt max among the PBS, 1 and 10 μg/kg rhHMGB1 groups (P>0.05, Table I). Injection of 100 μg/kg rhHMGB1 caused a significant deterioration in cardiac function (P<0.05, Table I).

In protocol 2, treatment with EP prior to reperfusion preserved the cardiac function. The LVSP, +dp/dt max and -dp/dt max were significantly improved in the EP group compared with the control group (P<0.05; Table I). When rhHMGB1 was coadministered with EP, the cardioprotective effect was blocked (Table I).

### Inflammatory cytokine expression following myocardial I/R

**Figure 2:**
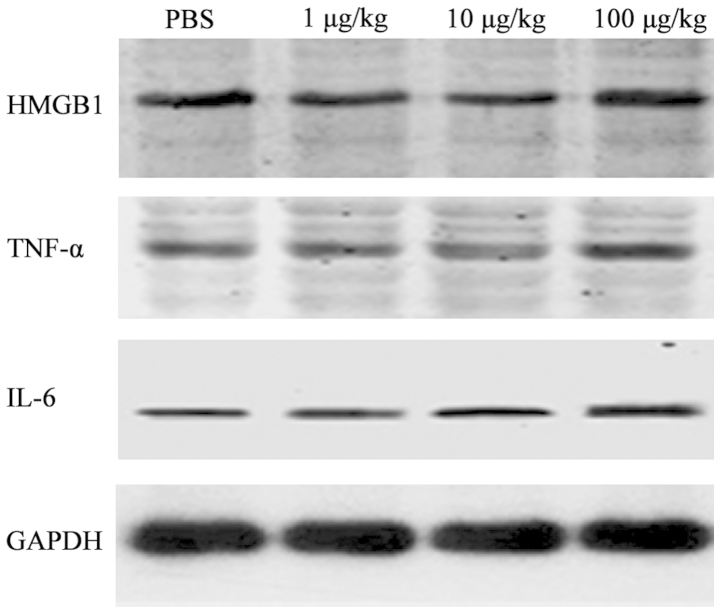
Effect of rhHMGB1 on the expression levels of HMGB1, TNF-α and IL-6 in a rat myocardial ischemia/reperfusion model. The expression levels of HMGB1, TNF-α, IL-6 and GAPDH were analyzed in the PBS and 1, 10 and 100 μg/kg rhHMGB1 groups. Western blot bands were quantified by densitometry. PBS, phosphate-buffered saline; rhHMGB1, recombinant human high-mobility group box-1; TNF-α, tumor necrosis factor-α; IL-6, interlukin-6; GAPDH, glyceraldehyde 3-phosphate dehydrogenase.

Western blotting was performed to assess the effect of rhHMGB1 on TNF-α and IL-6 expression. I/R resulted in a marked elevation in TNF-α and IL-6 levels. No significant increase in TNF-α or IL-6 was detected when 1 or 10 μg/kg rhHMGB1 was injected. When the dose of rhHMGB1 was increased to 100 μg/kg, the expression of TNF-α (0.65±0.06 vs. 0.47±0.05, P<0.05) and IL-6 (0.60±0.08 vs. 0.46±0.06, P<0.05) was markedly elevated compared with that in the PBS group (Fig. 2).

### EP inhibits HMGB1 and inflammatory cytokine expression following myocardial I/R

**Figure 3:**
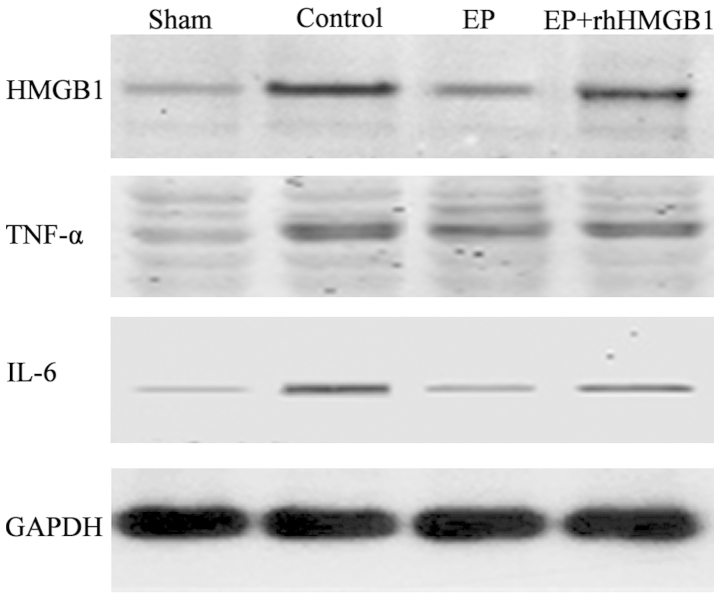
Effect of EP on the expression levels of HMGB1, TNF-α and IL-6 in a rat myocardial ischemia/reperfusion model. The expression levels of HMGB1, TNF-α, IL-6 and GAPDH were analyzed in the sham, control, EP and EP + HMGB1 groups. The EP group was administered EP at 40 mg/kg, while the EP + HMGB1 group was administered EP at 40 mg/kg and rhHMGB1 at 100 μg/kg. Western blot bands were quantified by densitometry. EP, ethyl pyruvate; rhHMGB1, recombinant human high-mobility group box-1; TNF-α, tumor necrosis factor-α; IL-6, interlukin-6; GAPDH, glyceraldehyde 3-phosphate dehydrogenase.

Compared with the sham group, HMGB1 expression was significantly increased in the control group 48 h after reperfusion (Fig. 3). Compared with the control group, administration of 40 mg/kg EP significantly inhibited the secretion of HMGB1 and decreased the inflammatory cytokine expression, but this protective effect was abrogated by the injection of rhHMGB1 (Fig. 3).

## Discussion

The pathogenesis of myocardial I/R injury is multifactorial (1). Proinflammatory cytokine release, endothelial cell activation and inflammatory cell infiltration are among the known factors that contribute to I/R injury. While the distal elements have been well characterized, the early molecular events that initiate I/R injury are poorly defined. HMGB1 can be passively released from damaged or necrotic cells and secreted by activated innate immune cells. Extracellular HMGB1 can trigger inflammation *in vitro* (12,13) and initiate and amplify inflammatory responses in non-infectious inflammatory conditions (14). In contrast to its delayed release following endotoxemia and microbial infection, HMGB1 is rapidly released subsequent to tissue I/R injury. Tsung *et al* (7) showed that HMGB1 levels were increased during liver I/R as early as 1 h after reperfusion and then increased in a time-dependent manner up to 24 h. In a mouse myocardial I/R model, Andrassy *et al* (6) reported that the mRNA and protein expression of HMGB1 increased after 30 min of ischemia and peaked at 6 h after reperfusion. In the present study in a rat *in vivo* model, the myocardial HMGB1 level increased significantly after 30 min ischemia and 48 h reperfusion. Strong expression of HMGB1 was observed on the infiltrating leukocytes in the ischemic area of the myocardium. Furthermore, the elevated HMGB1 level was positively correlated with the levels of myocardial TNF-α and IL-6. These data supported the findings of Andrassy *et al* (6) that HMGB1 is involved in the inflammatory response following I/R.

To further investigate the role of exogenous HMGB1 in a rat I/R model, an escalating dose of rhHMGB1 was injected intravenously prior to the onset of reperfusion. A previous study demonstrated that the administration of rHMGB1 worsened liver I/R injury (7). The deleterious effect of HMGB1 in I/R injury was also observed in the cardiovascular system. Furthermore, O’Connor *et al* (15) showed that intracerebroventricular injections of HMGB1 induced an increased level of IL-1 and impaired the central nervous system function in a dose-dependent manner (15). A single dose of 10 μg per mouse was selected in the study by Andrassy *et al* (6); however, the optimal dose for a rat model was unknown. In the current study, rhHMGB1 at various doses (1, 10 and 100 μg/kg) was injected to elucidate the effective dose in the rat myocardial I/R model. The doses of 1 and 10 μg/kg failed to induce further myocardial damage; however, administration of 100 μg/kg rhHMGB1 led to a significantly larger IS, elevated TNF-α and IL-6 levels and exacerbated the cardiac dysfunction.

Since HMGB1 acts as a central mediator of I/R injury, antagonism of HMGB1 may provide a novel therapeutic intervention. Investigations have shown that anti-HMGB1 treatment can ameliorate injury in multiple organ I/R models (6,16); however, HMGB1 antagonists (neutralizing monoclonal antibodies or HMGB1 box A) are expensive and require a complex technique.

EP, a stable and lipophilic derivative of pyruvate, was the first described pharmacological inhibitor for HMGB1 secretion (8). EP is readily available, and studies have demonstrated the protective effect of EP on the brain and kidney following I/R injury (17,18). Studies have also been conducted to investigate the cardioprotective effect of EP. Woo *et al* (19) showed that, in a rat I/R model, EP enhanced the myocardial adenosine triphosphate levels and attenuated myocardial oxidative injury. The anti-apoptosis effect was also found in an *in vitro* study (20). In the study by Woo *et al* (19), EP was injected prior to myocardial ischemia. It has previously been demonstrated that EP can afford strong protection of the spinal cord (21), even when it is administered 6 h after reperfusion. We speculated that EP could protect rats from myocardial I/R injury when it was administered just prior to the onset of reperfusion. This would be closer to the clinical condition, as myocardial infarction is usually unpredictable. In the current study, the intravenous injection of EP (40 mg/kg) before reperfusion significantly suppressed the elevated HMGB1 level. TNF-α and IL-6 expression levels are known to increase in the ischemic myocardium and play an important role in maintaining the inflammation cascade following reperfusion (22). Treatment with EP blocked the interaction between HMGB1 and the cytokines, reduced the TNF-α and IL-6 levels and preserved cardiac function. When exogenous HMGB1 was injected, the cardioprotective effect was abrogated. This indicated that the benefit of EP came from its inhibition of HMGB1.

In this rat *in vivo* I/R experiment, myocardial I/R resulted in a significant inflammatory response, accompanied by a strong upregulation of cardiac HMGB1, TNF-α and IL-6. Treatment with EP significantly attenuated cardiac I/R injury, and its protective effect was associated with decreases in the HMGB1 and local inflammatory cytokine levels. The beneficial effect of EP could be abrogated by rhHMGB1 injected prior to reperfusion. Thus, the current study revealed the potential role of EP as a therapeutic option for myocardial I/R.

## Figures and Tables

**Table I:** Hemodynamic function of rats 48 h after ischemia/reperfusion.

Group	HR (bmp/min)	LVSP (mmHg)	+dp/dt max (mmHg/sec)	−dp/dt max (mmHg/sec)
Protocol 1				
PBS	386±17	99±3	2890±217	1979±190
1 μg/kg HMGB1	394±11	97±3	2937±233	2007±219
10 μg/kg HMGB1	390±20	98±4	2744±261	2020±301
100 μg/kg HMGB1	389±20	85±3^a^	2260±156^a^	1588±119^a^
Protocol 2				
Sham	382±16	131±7	4674±282	3531±243
Control	395±17	101±5	2768±219	2102±155
EP	386±16	116±4^b^	3661±379^b^	2821±144^b^
EP + HMGB1	382±20	104±4^c^	3044±320^c^	2347±226^c^

The aim of protocol 1 was to determine the effect of various concentrations of rhHMGB1 on cardiac function; the aim of protocol 2 was to determine the effect of EP on cardiac function. Results are presented as the mean ± standard error; n=6–8 rats in all groups.

a P>0.05 vs. the PBS group;

b P<0.05 vs. the control group;

c P<0.05 vs. the EP group.

LVSP, left ventricular systolic pressure; HR, heart rate; +dp/dt max, maximum rate of left ventricular isovolumetric pressure development; -dp/dt max, maximum rate of left ventricular isovolumetric pressure fall; PBS, phosphate-buffered saline; EP, ethyl pyruvate; rhHMGB1, recombinant human high-mobility group box-1.
